# Ethanol production by the hyperthermophilic archaeon *Pyrococcus furiosus* by expression of bacterial bifunctional alcohol dehydrogenases

**DOI:** 10.1111/1751-7915.12486

**Published:** 2017-02-14

**Authors:** Matthew W. Keller, Gina L. Lipscomb, Diep M. Nguyen, Alexander T. Crowley, Gerrit J. Schut, Israel Scott, Robert M. Kelly, Michael W. W. Adams

**Affiliations:** ^1^ Department of Biochemistry and Molecular Biology University of Georgia Athens GA 30602 USA; ^2^ Department of Chemical and Biomolecular Engineering North Carolina State University Raleigh NC 27695 USA

## Abstract

Ethanol is an important target for the renewable production of liquid transportation fuels. It can be produced biologically from pyruvate, via pyruvate decarboxylase, or from acetyl‐CoA, by alcohol dehydrogenase E (AdhE). Thermophilic bacteria utilize AdhE, which is a bifunctional enzyme that contains both acetaldehyde dehydrogenase and alcohol dehydrogenase activities. Many of these organisms also contain a separate alcohol dehydrogenase (AdhA) that generates ethanol from acetaldehyde, although the role of AdhA in ethanol production is typically not clear. As acetyl‐CoA is a key central metabolite that can be generated from a wide range of substrates, AdhE can serve as a single gene fuel module to produce ethanol through primary metabolic pathways. The focus here is on the hyperthermophilic archaeon *Pyrococcus furiosus,* which grows by fermenting sugar to acetate, CO_2_ and H_2_. Previously, by the heterologous expression of *adhA* from a thermophilic bacterium, *P. furiosus* was shown to produce ethanol by a novel mechanism from acetate, mediated by AdhA and the native enzyme aldehyde oxidoreductase (AOR). In this study, the AOR gene was deleted from *P. furiosus* to evaluate ethanol production directly from acetyl‐CoA by heterologous expression of the *adhE* gene from eight thermophilic bacteria. Only AdhEs from two *Thermoanaerobacter* strains showed significant activity in cell‐free extracts of recombinant *P. furiosus* and supported ethanol production *in vivo*. In the AOR deletion background, the highest amount of ethanol (estimated 61% theoretical yield) was produced when *adhE* and *adhA* from *Thermoanaerobacter* were co‐expressed.

## Introduction

Currently, ethanol is by far the most common biofuel, and there are three metabolic starting points for biological ethanol production: pyruvate, acetyl‐CoA and acetate. These metabolic intermediates are converted to acetaldehyde, which can then be converted to ethanol via an alcohol dehydrogenase. The most common pathway for ethanol production in mesophilic microorganisms involves pyruvate decarboxylation to acetaldehyde and subsequent reduction to ethanol, via pyruvate decarboxylase (PDC) and an alcohol dehydrogenase (ADH; Hoppner and Doelle, 1983). Extremely thermophilic bacteria lack PDC, and these utilize the bifunctional aldehyde dehydrogenase (ALDH)/alcohol dehydrogenase (ADH), also known as alcohol dehydrogenase E (AdhE), to perform the two step reduction of acetyl‐CoA to ethanol. Acetyl‐CoA is an attractive substrate for formation of fuels such as ethanol, because it is a central metabolite (Nikolau *et al*., 2000), and can serve as a link between fuel production and a variety of substrate utilization pathways. The bifunctional AdhE is widely found across Bacteria and Eukarya, from the thermophilic strictly anaerobic bacterium *Thermoanaerobacter ethanolicus* (Lee *et al*., 1993) to the green alga *Chlamydomonas reinhardtii* (Shrager *et al*., 2003); however, it is conspicuously absent from members of the Archaeal domain.

There is growing interest in thermophilic routes for biofuel production, as industrial‐scale high temperature fermentation has the advantages of reduced risk of contamination, lower cooling costs and the potential for continuous removal of volatile products (Taylor *et al*., 2009; Abdel‐Banat *et al*., 2010; Zeldes *et al*., 2015). AdhE has played a central role in increasing the maximum temperature for metabolically engineered ethanol production, as thermostable versions of pyruvate decarboxylase have not been discovered (Olson *et al*., 2015). Bacteria with either native or heterologous AdhE genes have been engineered for ethanol production at higher temperatures. For example, extensive mutational and overexpression studies have improved ethanol tolerance and production in *Clostridium thermocellum* and *Thermoanaerobacterium saccharolyticum* at 55°C (Zheng *et al*., 2015). In *G. thermoglucosidasius*, the deletion of lactate dehydrogenase (LDH), formate dehydrogenase (FDH) and overexpression of pyruvate dehydrogenase (PDH) directed carbon and electron flow to improve ethanol production at 60°C (Cripps *et al*., 2009). The overexpression of *Clostridium thermocellum* AdhE in a *Caldicellulosiruptor bescii* strain with a disrupted LDH resulted in ethanol production from plant biomass at 65°C (Chung *et al*., 2014). *Thermoanaerobacter mathranii* lactate dehydrogenase (LDH) was knocked out, and its AdhE was overexpressed, resulting in an improvement in ethanol production at 70°C (Yao and Mikkelsen, 2010a,b). *C. bescii* has also been engineered with the AdhE from *Thermoanaerobacter pseudethanolicus* 39E, which grows optimally at 65°C (Onyenwoke *et al*., 2007), and this supported ethanol production at 75°C (Chung *et al*., 2015).

**Figure 1:**
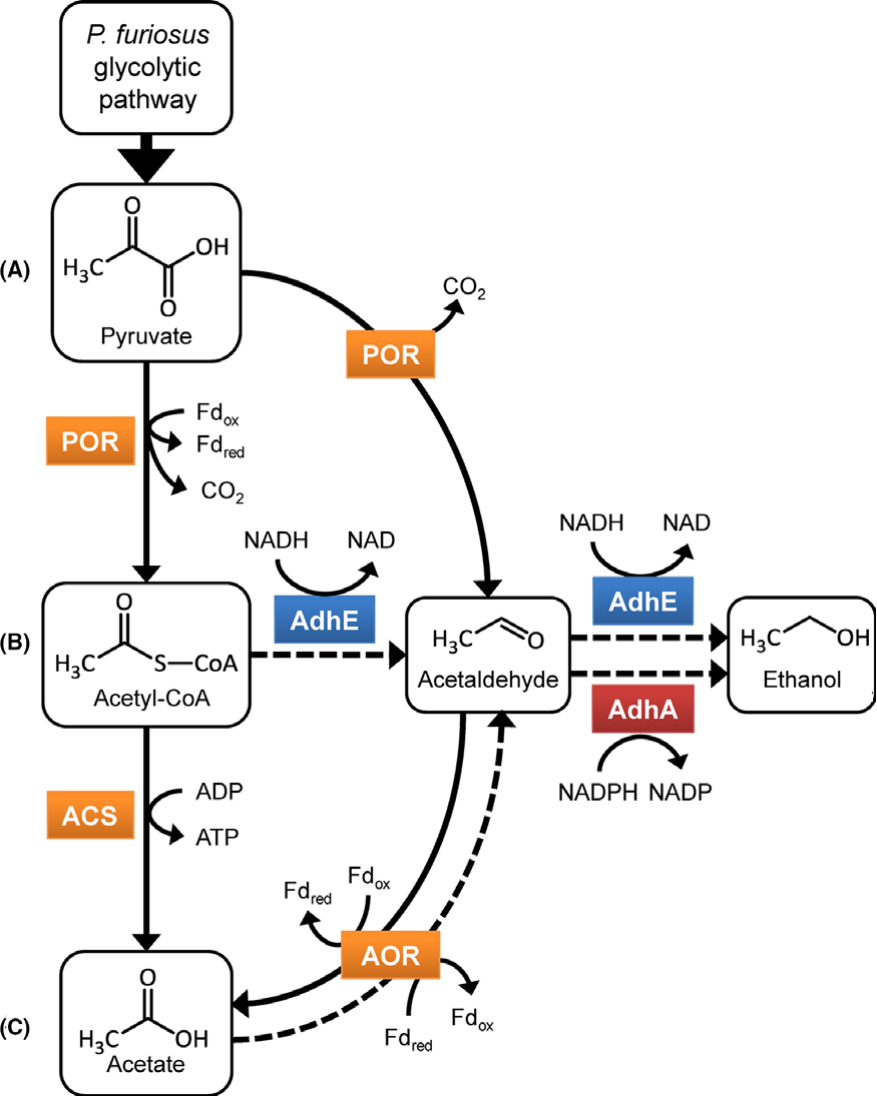
Pathways for native and engineered ethanol production in *Pyrococcus furiosus*. Glycolysis to pyruvate (0.5 glucose) yields no ATP and produces 2 Fd_red_ (net 2 e^−^). Ethanol production from: (A) pyruvate via the pyruvate decarboxylase activity of pyruvate ferredoxin oxidoreductase (POR) and an alcohol dehydrogenase (ADH) results in no net ATP, produces 2 Fd_red_ and uses 1 NAD(P)H (net 2 e^−^); (B) acetyl‐CoA via AdhE yields no net ATP, produces 4 Fd_red_ and uses 2 NADH (net 0 e^−^); (C) acetate via aldehyde ferredoxin oxidoreductase (AOR, here also representing other oxidoreductases with aldehyde oxidizing activity), and AdhA results in 1 net ATP via acetyl‐CoA synthase (ACS), produces 2 Fd_red_ and uses 1 NADPH (net 0 e^−^). *P. furiosus* enzymes are shown in orange, AdhA is shown in red, and AdhE is shown in blue. Proposed physiological reactions are indicated with solid arrows, and engineered pathways in recombinant strains are indicated with dashed arrows.

A pathway for ethanol production from acetate was recently demonstrated in the thermophilic archaeon *Pyrococcus furiosus* (Roh *et al*., 2002; Basen *et al*., 2014). *P. furiosus* metabolizes carbohydrates at temperatures near 100°C to primarily H_2_, CO_2_ and acetate (Fiala and Stetter, 1986; Kengen *et al*., 1996). Heterologous expression of the AdhA gene from *Thermoanaerobacter* sp. strain X514 (*T. *X514) in *P. furiosus* allowed for ethanol production at temperatures up to 78°C (Basen *et al*., 2014; Nguyen *et al*., 2015). As shown in Fig. 1, the acetaldehyde substrate for AdhA is generated by the ferredoxin‐dependent aldehyde oxidoreductase (AOR) of *P. furiosus*. This enzyme is thought to oxidize acetaldehyde for detoxification purposes (Basen *et al*., 2014). However, in the presence of AdhA, AOR catalyses the reverse reaction, acetate reduction, and drives the AdhA‐catalysed reduction of acetaldehyde to ethanol. In *P. furiosus,* the acetate is generated from acetyl‐CoA by acetyl‐CoA synthetase (ACS), which also generates ATP (Fig. 1).

Hence, in *P. furiosus,* a combination of ACS, AOR and AdhA, to be referred to here as the AAA pathway, catalyses the same overall reaction as AdhE, that is, the conversion of acetyl‐CoA to ethanol (Fig. 1).

In this study, AdhE genes from eight thermophilic bacteria were selected for expression in a strain of *P. furiosus* lacking the AOR gene to examine the ethanol production capabilities of *P. furiosus* from only acetyl‐CoA and not acetate. The AdhE activities in cell extracts were examined as was the ability of the recombinant strains to produce ethanol. In addition, as the AdhE genes are from bacteria with optimal growth temperatures significantly lower than that of *P. furiosus*, which grows optimally near 100°C, a temperature shift strategy was used for their expression. The results here demonstrate *in vitro* AdhE‐dependent catalytic activity and *in vivo* ethanol production for two of eight thermophilic AdhE homologs expressed in *P. furiosus*.

## Results

### Selection of AdhE source organisms

**Figure 2:**
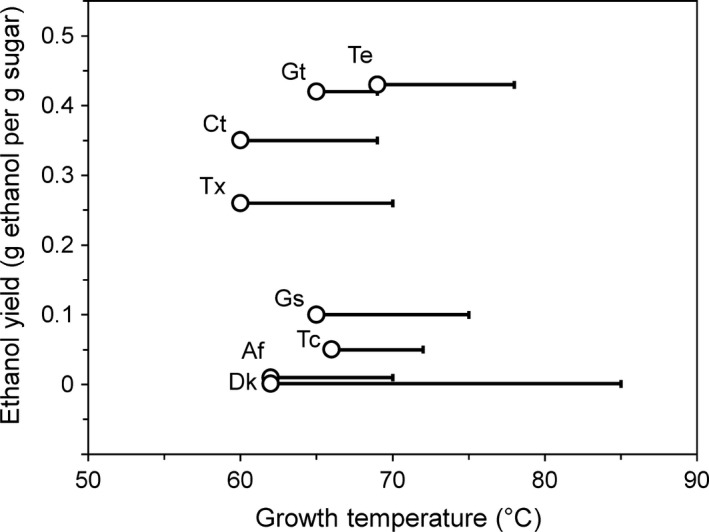
Ethanol yield versus growth temperature of selected source organisms for AdhE genes. Organism abbreviations are listed in Table 1. Optimal growth temperature is indicated by an open circle with a line indicating the maximum growth temperature. Ethanol yields were calculated from previously published data (Lamed and Zeikus, 1980; Bryant *et al*., 1992; Engle *et al*., 1996; Peng *et al*., 2008; Cripps *et al*., 2009; Dai *et al*., 2011; Hemme *et al*., 2011; Lo *et al*., 2015). Dk is reported to grown on but not produce ethanol (Visser *et al*., 2013).

*Pyrococcus furiosus* is capable of growth at temperatures as low as 70°C, almost 30°C below its optimal growth temperature (Weinberg *et al*., 2005). At temperatures below 70°C, *P. furiosus* metabolic processes continue, as shown in previous work in strains engineered for butanol formation, which was demonstrated at temperatures as low as 60°C (Keller *et al*., 2015). In choosing AdhE‐encoding genes for expression in *P. furiosus*, candidate organisms were selected based on a minimum growth temperature of 60°C. The AdhE gene from *Clostridium thermocellum*, which grows optimally at 60°C, has been heterologously expressed in a thermophilic bacterium producing up to 15 mM ethanol at 65°C (Chung *et al*., 2014). We therefore used the *C. thermocellum* AdhE gene to perform a BLAST search (Altschul *et al*., 1990) against the NCBI database, including the search term ‘therm*’ to limit hits to thermophilic organisms (by definition, organisms that have optimal growth temperatures at 45°C and above). Of the approximately 60 resulting hits, a total of eight bacteria that had optimal growth temperatures of 60°C or higher were selected, including two representatives each of *Geobacillus* and *Thermoanaerobacter* species, along with *C. thermocellum* (Table 1). There were no AdhE homologs in any archaeon, regardless of growth temperatures. The eight AdhE enzymes had 46–63% sequence identity (67–80% similarity) to *E. coil* AdhE, with various degrees of sequence homology within the group (Table S1). Many of these organisms naturally produce ethanol, with *T. ethanolicus* having both the highest growth temperature optimum and the highest reported ethanol yield (Fig. 2). The AdhE enzymes from some of these organisms have been characterized *in vitro*, including recombinant forms of TxAdhE (Loder *et al*., 2015), CtAdhE (Loder *et al*., 2015) and TeAdhE (Peng *et al*., 2008), as well as natively purified GtAdhE (Extance *et al*., 2013).

**Table 1:** Source organisms for AdhE genes

Abbr.	Organism	Growth optimum (range; °C)	Genome GC content (%)	AdhE GenBank Accession	AdhE sequence identity to Ct (%)
Ct	*Clostridium thermocellum* (ATCC 27405)	60 (28–69)	39	WP_003519039	100
Tx	*Thermoanaerobacter* sp. X514	60 (50–70)	34.5	ABY91935	52
Gt	*Geobacillus thermoglucosidasius* NBRC 107763	62 (42–69)	43.7	GAJ43611	51
Dk	*Desulfotomaculum kuznetsovii* DSM 6115	62.5 (50–85)	54.9	WP_013822371	47
Af	*Anoxybacillus flavithermus* WK1	62.5 (40–70)	41.8	ACJ35167	51
Gs	*Geobacillus stearothermophilus* NUB3621	65 (48–73)	44.4	EZP74770	51
Tc	*Thermobrachium celere* DSM 8682	66 (43–75)	31.3	WP_018660634	71
Te	*Thermoanaerobacter ethanolicus* JW200	69 (37–78)	34.1	WP_003868676	52

### Strain construction and validation

There is a robust genetic system available for *P. furiosus*. This utilizes a single counter‐selectable marker (*pyrF*) in a naturally competent genetic background strain having a deletion of the *pyrF* gene (COM1), allowing multiple deletions and/or insertions to be made in a single strain (Lipscomb *et al*., 2011; Farkas *et al*., 2012). To facilitate analysis of ethanol produced exclusively as a result of heterologous expression of *adhE*, a background strain lacking *aor* was constructed in COM1 to eliminate ethanol production from acetate‐derived acetaldehyde (Basen *et al*., 2014). Deletion of the AOR gene was previously demonstrated to reduce ethanol production in an AdhA‐expressing strain to background levels (Basen *et al*., 2014). The deletion of AOR did not affect growth on maltose and resulted in an 80% decrease in acetaldehyde‐dependent benzyl viologen (BV) reduction activity (Fig. S2). The remaining 20% of activity can be attributed to other AOR family enzymes that also display some activity with acetaldehyde (Roy *et al*., 1999; Roy and Adams, 2002; Bevers *et al*., 2005). The Δ*aor* genetic background was used for expression of AdhE constructs placed under control of the promoter of the highly expressed S‐layer protein gene (P_*slp*_) (Keller *et al*., 2013) and inserted between genes PF0738 and PF0739, a location referred to here as Genome Region 4. Eight strains contained the AdhE from each organism listed in Table 1. An additional strain containing the *T. ethanolicus* AdhE in a synthetic operon with AdhA was also constructed. The AdhA gene was amplified from *T. *X514; it should be noted that the amino acid sequence of TxAdhA is identical to that of TeAdhA. The previously published strain MW611, containing a deletion of *aor* in the AdhA expression strain (Basen *et al*., 2014), was also used for comparison. The control strains used in growth experiments were the COM1c2 strain containing *pyrF* added back to COM1 to restore uracil prototrophy (Thorgersen *et al*., 2012), and the ΔAORc strain containing a marker‐replaced deletion of *aor*. The strains used as controls in the activity assays were the uracil auxotrophic versions of these strains, COM1 and ΔAOR. All *P. furiosus* strains used and constructed in this study are listed in Table 2.

**Table 2:** *Pyrococcus furiosus* strains used and constructed in this study

Strain	Alias	Genotype^a^	Reference
*Genetic background and control strains*			
MW002	COM1	Δ*pyrF*	Lipscomb *et al*. (2011)
MW252	ΔAOR	Δ*pyrF* Δ*aor*	This work
MW004	COM1c2	Δ*pyrF*::*pyrF*	Thorgersen *et al*. (2012)
MW616	ΔAORc	Δ*pyrF* Δ*aor*::P_*gdh*_ *pyrF*	This work
MW611	AdhA	Δ*pyrF* GR3::P_*slp*_Tx*adhA* Δ*aor*::P_*gdh*_ *pyrF*	Basen *et al*. (2014)
*AdhE expression strains*			
MW627	Ct‐AdhE	Δ*pyrF* Δ*aor* GR4::P_*slp*_ Ct*adhE* P_*gdh*_ *pyrF*	This work
MW332	Tx‐AdhE	Δ*pyrF* Δ*aor* GR4::P_*slp*_ Tx*adhE* P_*gdh*_ *pyrF*	This work
MW371	Gt‐AdhE	Δ*pyrF* Δ*aor* GR4::P_*slp*_ Gt*adhE* P_*gdh*_ *pyrF*	This work
MW364	Dk‐AdhE	Δ*pyrF* Δ*aor* GR4::P_*slp*_ Dk*adhE* P_*gdh*_ *pyrF*	This work
MW373	Af‐AdhE	Δ*pyrF* Δ*aor* GR4::P_*slp*_ Af*adhE* P_*gdh*_ *pyrF*	This work
MW357	Gs‐AdhE	Δ*pyrF* Δ*aor* GR4::P_*slp*_ Gs*adhE* P_*gdh*_ *pyrF*	This work
MW372	Tc‐AdhE	Δ*pyrF* Δ*aor* GR4::P_*slp*_ Tc*adhE* P_*gdh*_ *pyrF*	This work
MW392	Te‐AdhE	Δ*pyrF* Δ*aor* GR4::P_*slp*_ Te*adhE* P_*gdh*_ *pyrF*	This work
MW325	Te‐AdhEA	Δ*pyrF* Δ*aor* GR4::P_*slp*_ Te*adhE* Tx*adhA* P_*gdh*_ *pyrF*	This work
*Mutant AdhE expression strains*			
MW623	Tx‐AdhE*	Δ*pyrF* Δ*aor* GR4::P_*slp*_ Tx*adhE*(G557D) P_*gdh*_ *pyrF*	This work
MW625	Te‐AdhE*	Δ*pyrF* Δ*aor* GR4::P_*slp*_ Te*adhE*(G532D) P_*gdh*_ *pyrF*	This work

a GR3 represents insertion at genome region 3 (between genes PF0574 and PF0575), and GR4 represents insertion at genome region 4 (between genes PF PF0738.1n and PF0739). Organism abbreviations are listed in Table 1.

### In vivo ethanol production of AdhE‐containing strains

**Figure 3:**
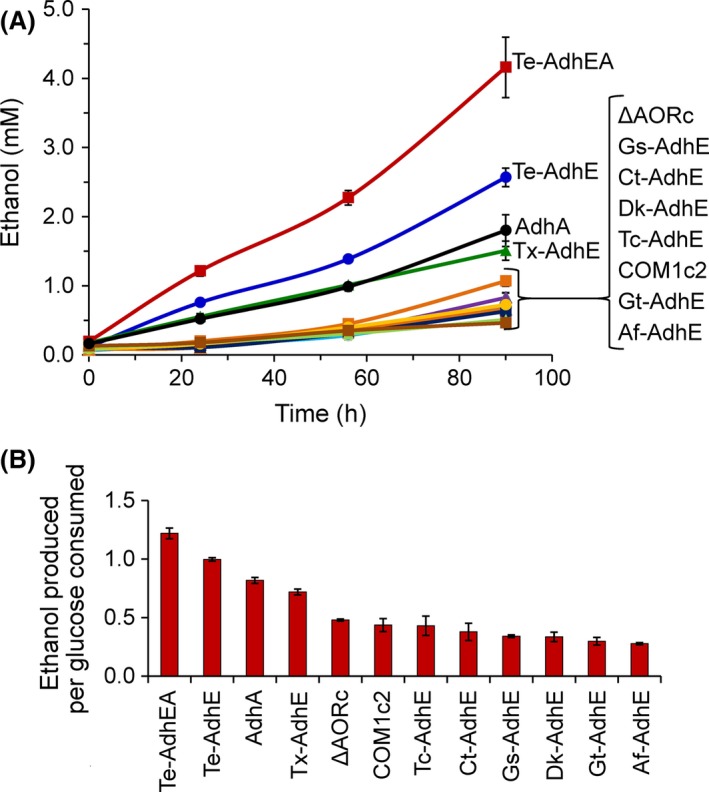
Ethanol production by AdhE‐containing strains of *Pyrococcus furiosus*. All strains, aside from COM1c2, have AOR deleted. *P. furiosus* strains and two control strains (ΔAORc and COM1c2) were grown in closed serum bottles with 50 ml YM5 medium to mid‐log phase at 95°C and then incubated at 65°C for 90 h. A. Ethanol production over time. B. Ethanol produced per estimated glucose consumed, calculated from ethanol and acetate values for the 90 h time point (shown in A), ordered from highest to lowest. Ethanol and acetate are assumed to be the only carbon end‐products after glycolysis. Error bars represent SD, *n *=* *3.

As the AdhE enzymes were obtained from organisms with optimal growth temperatures ranging from 60 to 70°C, and *P. furiosus* grows optimally at near 100°C, a strategy involving a temperature shift was used for growth to measure *in vivo* ethanol production. All strains were grown at 95°C to mid‐log phase (1 × 10^8^ cells ml^−1^), and cultures were then shifted to 65°C and incubated for up to 90 h for ethanol production (Fig. 3A). A low concentration of ethanol (≤ 1 mM) was observed in the control strains COM1c2 and ΔAOR, consistent with previous results (Basen *et al*., 2014). No ethanol production was observed at 95°C from any strains prior to the temperature shift (data not shown).

Of the bacterial strains expressing the various heterologous AdhE genes, only those containing AdhE and AdhA from *Thermoanaerobacter* sp. produced ethanol above background (Fig. 3A). The Te‐AdhEA strain containing both AdhE and AdhA produced the most ethanol (4.2 mM), followed by Te‐AdhE (2.6 mM), AdhA (1.8 mM) and Tx‐AdhE (1.5 mM). Acetoin has recently been reported as a major fermentation end‐product of *P. furiosus* when grown at 70–80°C (Nguyen *et al*., 2015); however, under these conditions at 65°C, no acetoin was detected. Therefore, ethanol and acetate are the only major carbon end‐products from glucose under these conditions. For these four strains, the amount of ethanol produced per estimated glucose consumed was increased from the background level (~0.3–0.4) to 1.2, 1.0, 0.8 and 0.7 respectively (Fig. 3B).

### Construction and analysis of strains expressing mutated AdhE genes

It was considered that nucleotide specificity of the ADH enzymes might play a role in ethanol yield *in vivo*, as AdhE is NADH‐dependent. Under physiological conditions (where [NADPH] > [NADP]), the reduction potential of NADPH (approx. –380 mV) is lower than that of NADH (~ –280 mV, [NADH] < [NAD] (Huang *et al*., 2012)). In *P. furiosus*, NADPH can be generated from fermentatively produced H_2_ via soluble hydrogenase I (Mertens *et al*., 2003). There are a number of reports that identify mutations in AdhE that cause a change in nicotinamide nucleotide specificity. For example, a glycine to aspartic acid mutation in the NADH binding site of the ADH domain of AdhE from *Thermoanaerobacterium saccharolyticum* (G544D in strains LL1049 and LL1194) results in a switch from NADH to NADPH, without any dramatic reduction in enzyme activity. Interestingly, this switch in specificity is observed for both ALDH and ADH activities, even though the mutation is located in the ADH domain (Zheng *et al*., 2015). The amino acid sequence of *T*. *saccharolyticum* AdhE is 86% and 87% identical (93% and 94% similar) to TeAdhE and TxAdhE respectively. Therefore, these mutations were introduced into both *Thermoanaerobacter* AdhE genes, and they were each expressed in a Δ*aor P. furiosus* background. Unfortunately, these mutations did not result in NADPH‐dependent ALDH or ADH activities in cell extracts; rather, they increased the NADH‐dependent ALDH activities, but abolished the NADH‐dependent ADH activities (Table 3). Rather than improving ethanol yield, the mutations had the effect of reducing ethanol production to the background level (Fig. S2).

**Table 3:** ALDH‐ and ADH‐specific activities in *Pyrococcus furiosus* strains and bacterial crude extracts

Strain	ALDH activity^a^ (acetyl‐CoA)		ADH activity^a^ (acetaldehyde)	
	NADH	NADPH	NADH	NADPH
*Control strains*				
COM1	< 0.05	< 0.05	< 0.05	< 0.05^b^
ΔAOR	< 0.05	< 0.05	< 0.05	< 0.05^b^
AdhA	< 0.05	< 0.05	< 0.05	0.94
*P. furiosus AdhE expression strains*				
Te‐AdhEA	0.58	< 0.05	0.48	1.66
Te‐AdhE	0.29	< 0.05	1.00	0.07
Tx*‐*AdhE	0.17	< 0.05	0.53	< 0.05
Gt‐AdhE	< 0.05	< 0.05	< 0.05	< 0.05
Dk‐AdhE	< 0.05	< 0.05	0.05	< 0.05
Af‐AdhE	< 0.05	< 0.05	0.05	< 0.05
Gs‐AdhE	< 0.05	< 0.05	< 0.05	< 0.05
Tc‐AdhE	< 0.05	< 0.05	0.06	< 0.05
Ct‐AdhE	< 0.05	< 0.05	< 0.05	< 0.05
*Mutant AdhE expression strains*				
Te‐AdhE*	0.44	< 0.05	< 0.05	< 0.05
Tx‐AdhE*	0.72	< 0.05	< 0.05	< 0.05
*AdhE in the native organism*				
*C. thermocellum*	4.96	0.06	19.42	< 0.05
*T*. sp. strain X514	0.18	0.02	1.09	3.38
*T. ethanolicus*	0.70	< 0.05	2.82	6.96

a Specific activities are listed as μmol NAD(P)H oxidized min^−1^ mg^−1^. The detection limit for these assays is ~0.05 U mg^−1^.

b ADH activity in the forward reaction was not detected at 65°C; however, the reverse assay resulted in 0.2 U mg^−1^ of ethanol oxidation using NADP as the electron acceptor at 80°C.

### In vitro ALDH and ADH activities in P. furiosus AdhE‐containing strains

To determine the levels of activity of the various heterologously expressed AdhE and AdhA enzymes in *P. furiosus*, cultures were grown to mid‐log phase at 95°C and then switched to 65°C for 40 h. Cell‐free extracts were assayed anaerobically at 65°C for ALDH activity (NADH‐ or NADPH‐dependent reduction of acetyl‐CoA) and ADH activity (NADH‐ or NADPH‐dependent reduction of acetaldehyde).

AdhE is a bifunctional enzyme, containing both ALDH and ADH activities, both of which have been reported to be NADH‐dependent in previously characterized AdhE enzymes (Kessler *et al*., 1991). NADH‐dependent ALDH activity was found in strains expressing the wild‐type or mutated *Thermoanaerobacter* AdhE enzymes, and these values were comparable to those measured in cell extracts of the source organisms (Table 3) and were about an order of magnitude lower than those measured from purified recombinant TeAdhE (Peng *et al*., 2008). No NADPH‐dependent ALDH activity was detected. For Tx‐AdhE, Te‐AdhE and Te‐AdhEA strains, which contained wild‐type AdhE enzymes, extracts contained NADH‐specific ADH activity. Strains AdhA and Te‐AdhEA also displayed high NADPH‐dependent ADH activity due to the AdhA, which has been previously shown to be highly active and NADPH‐specific (Basen *et al*., 2014). In the strains containing mutated *Thermoanaerobacter* AdhE enzymes (Tx‐AdhE* and Te‐AdhE*), the ADH activity was abolished. For the remaining AdhE‐containing strains, no ALDH activity and very little, if any, ADH activity was detected (Table 3). For *P. furiosus* genetic background strains COM1 and ΔAOR, no activity above the detection limit (~0.05 U mg^−1^) was observed for the following reactions: the reduction of acetyl‐CoA via NAD(P)H, the reduction of acetaldehyde via NAD(P)H or the oxidation in ethanol via NADH or BV. However, the cell extracts of both control strains contained 0.2 ± 0.05 U mg^−1^ of NADP‐dependent ethanol oxidation at 80°C.

## Discussion

There are three possible routes of either native or engineered ethanol production in *P. furiosus*, stemming from three different sources of acetaldehyde as an intermediate: pyruvate, acetyl‐CoA and acetate (Fig. 1). Previous work demonstrated that the native AOR enzyme can function in concert with a heterologously expressed AdhA enzyme to drive ethanol production from acetate (Basen *et al*., 2014), referred to here as the AAA pathway. We show here that *P. furiosus* also has the capability for ethanol production from acetyl‐CoA catalysed by AdhE (Fig. 1).

Two of eight AdhE genes expressed in *P. furiosus* were functional, despite the substantial sequence identity (47–97%, with 67–98% similarity) among all of the AdhE proteins (Table S1). The strains containing the *Thermoanaerobacter* AdhE enzymes, Te‐AdhE and Tx‐AdhE, produced ethanol *in vivo* (Fig. 3) and contained measurable NADH‐dependent ALDH activity (Table 3 and Fig. 3). Expressing AdhA along with AdhE from *T. ethanolicus* (strain Te‐AdhEA) resulted in the highest concentration of ethanol measured (1.2 mole ethanol produced per estimated mole glucose consumed). The improvement in ethanol yield with Te‐AdhEA compared to Te‐AdhE indicates that the highly active AdhA augments the AdhE pathway by providing an NADPH‐specific route to ethanol from acetaldehyde. Specifically, the contribution of AdhA to this AdhE strain results in an increase in 1.6 mM in ethanol production (an additional 0.2 mole ethanol per estimated mole glucose). Interestingly, the control strain expressing AdhA alone, with AOR deleted, resulted in similar ethanol production as the Tx‐AdhE strain (an increase of ~0.7 mM over the background concentration), suggesting that AdhA is highly efficient for scavenging acetaldehyde. While it is clear that AdhA can produce ethanol from natively generated acetaldehyde, it appears that AdhA also directly augments the AdhE pathway by utilizing acetaldehyde produced as an intermediate by AdhE, as is the case with ethanologenesis in *Thermoanaerobacter mathranii* (Yao and Mikkelsen, 2010b). The ethanol‐boosting effect of AdhA on AdhE is further supported by *in vitro* results showing that purified TxAdhE has a high *Km* for acetaldehyde (22 ± 4.8 mM (Loder *et al*., 2015)), given that TxAdhA has a much lower *Km* for acetaldehyde (63 μM (Basen *et al*., 2014)). In a previous study, a similar effect was seen in an AOR‐containing background where, at 72°C, a strain containing TxAdhE produced 2 mM ethanol and a strain with both TxAdhE and AdhA produced 10 mM ethanol (Basen *et al*., 2014). However, most of the ethanol in those strains was likely derived from acetate (via AOR), rather than from acetyl‐CoA. Growth of *P. furiosus* at suboptimal temperatures from 70 to 80°C can result in the production of acetoin, in addition to acetate, H_2_ and CO_2_ (Nguyen *et al*., 2015). However, we did not observe acetoin production in our strains at 65°C. Therefore, assuming that ethanol or acetate is the main carbon end‐products after glycolysis, strain Te‐AdhEA produced ethanol from glucose at an estimated 61% theoretical yield (Fig. 3B).

The selected expression temperature (65°C) for the heterologous *P. furiosus* strains was within the growth temperature ranges of all of the source bacteria (Fig. 2). Yet, only the two *Thermoanaerobacter* AdhE enzymes were functional in *P. furiosus*. Although these were from the upper and lower end of the optimal growth temperatures (Table 1), the two enzymes share 97% sequence identity (Table S1). Clearly, growth temperature of the source organisms is not a sufficient indicator of biocatalytic function *in vivo* in *P. furiosus*. Other factors that could influence heterologous enzyme function in *P. furiosus* include genetic characteristics such as codon usage. Although the source organisms have similar GC content to that of *P. furiosus* (40.8%; Table 1), slight differences in codon usage, particularly in higher GC content organisms, could affect enzyme expression. In addition, all of the bacteria were isolated from terrestrial locations, while *P. furiosus* is a marine organism that is known to accumulate a range of intracellular solutes (Santos and Da Costa, 2002); this factor might affect the functionality of the heterologously produced enzymes. The effect of nucleotide specificity of the AdhE on ethanol production in *P. furiosus* could not be investigated as specific mutations in the *Thermoanaerobacter* AdhEs did not affect a change from NADH to NADPH specificity, contrary to what was reported in literature for the highly homologous *T. saccharolyticum* AdhE (Zheng *et al*., 2015). Additionally, AdhE has been reported to form large multimeric complexes termed spirosomes, which are thought to enhance stability or facilitate channelling of the acetaldehyde intermediate (Kessler *et al*., 1991; Bruchhaus and Tannich, 1994; Extance *et al*., 2013), and expression of these enzymes outside their native context may disrupt formation of these structures. The metabolic context of the host organism is clearly important, as even though heterologous expression of *C. thermocellum* AdhE in *C. bescii* resulted in ~10 mM ethanol produced at 65°C (Chung *et al*., 2014), this AdhE did not lead to activity or increased ethanol production in *P. furiosus* at that temperature.

The present study has shed some light on possible routes for native ethanol production in *P. furiosus* (Fig. 1). At suboptimal temperatures, *P. furiosus* produces a small amount (~1 mM) of ethanol (Basen *et al*., 2014), which we also observed in this study (Fig. 3). *P. furiosus* contains a number of alcohol dehydrogenase genes, some of which have been characterized (van der Oost *et al*., 2001; Machielsen and van der Oost, 2006). These ADHs are likely responsible for reducing natively produced acetaldehyde to ethanol (Fig. 1). Interestingly, deletion of the AOR gene in *P. furiosus* strain COM1 did not abolish the ethanol that was produced at 65°C. Both the parent and *aor* deletion strains produced 0.3 mole ethanol per mole of acetate, or an estimated 23% of the theoretical yield from glucose (Fig. 3B). Therefore, AOR plays little if any role in natural *P. furiosus* ethanol production. Interestingly, cell extracts of the AOR deletion strain contained ~20% of the dye‐linked acetaldehyde oxidoreductase activity of the parent strain (Fig. S1), indicating that other enzymes are present that could reduce acetate to acetaldehyde. These include the other members of the tungsten‐containing AOR family that are present in *P. furiosus,* including formaldehyde ferredoxin oxidoreductase (FOR) (Roy *et al*., 1999) and two other partially characterized tungsten‐containing oxidoreductases (WOR4 and WOR5) (Roy and Adams, 2002; Bevers *et al*., 2005). In addition, acetaldehyde is produced as a side reaction of pyruvate ferredoxin oxidoreductase (Ma *et al*., 1997), which converts pyruvate to acetyl‐CoA (Fig. 1). Furthermore, in the absence of *aor*, expression of AdhA alone results in a ~0.7 mM increase in ethanol production, confirming the presence of other acetaldehyde‐producing enzymes in *P. furiosus*.

Although ethanol production from acetyl‐CoA was demonstrated in *P. furiosus* in this study, the highest ethanol yield (from strain Te‐AdhEA) is still lower than that of the previously reported AAA pathway in *P. furiosus*, which functions via native enzymes ACS and AOR along with heterologously expressed AdhA. Ethanol production in strain MW608, expressing AdhA and containing intact AOR, resulted in ~1.8 mole ethanol produced per mole estimated glucose consumed which is approximately 90% theoretical yield, assuming ethanol and acetate are the only two carbon end‐products after glucose fermentation (Basen *et al*., 2014). A distinct advantage of the AAA pathway is the ATP‐generating step converting acetyl‐CoA to acetate. Furthermore, reducing equivalents for the pathway ultimately are derived from ferredoxin generated during glycolysis. Acetate to acetaldehyde conversion by AOR is ferredoxin‐dependent, and AdhA utilizes NADPH which can be regenerated by Soluble Hydrogenase I (SHI) via uptake of H_2_ generated by the ferredoxin‐dependent membrane bound hydrogenase (MBH; Mertens *et al*., 2003). The AAA pathway is therefore well integrated with *P. furiosus* glycolysis in terms of energy conservation and redox balance. Conversely, the AdhE pathway relies solely on NADH (unless AdhA is also expressed), and it is unclear if and how the pools of NADH and ferredoxin are interconverted in *P. furiosus*, although a possible enzyme candidate is the NADH dependent NADP:ferredoxin oxidoreductase I (NfnI), formerly known as the ferredoxin‐NAD(P)H oxidoreductase I (FNORI) (Ma and Adams, 2001). Furthermore, AdhE diverts acetyl‐CoA away from the energy conserving ACS reaction. At first glance, the AdhE enzymes appear to function in a very predictable manner; however, it is becoming clear that their characteristics are very intricate, and engineering pathways that contain these enzymes to function in a heterologous host is not a straight‐forward task. A better understanding of how these enzymes work in their respective native contexts would benefit future efforts to utilize them for applied purposes.

## Experimental procedures

### Selection of AdhE gene source organisms

The *C. thermocellum* AdhE gene was used to perform a blast search (Altschul *et al*., 1990) against the NCBI database including the search term ‘therm*’. Out of the approximately 60 resulting hits, seven organisms in addition to *C. thermocellum* were selected based on their growth temperature profile (Table 1). Organisms or genomic DNA were obtained from the Leibniz Institute DSMZ German Collection of Microorganisms and Cell Cultures (Braunschweig, Germany) for the following strains: *T. ethanolicus* JW200 (DSM 2246), *G. thermoglucosidasius* NBRC 107763 (DSM 2542), *T. celere* (DSM 8682), *A. flavithermus* WK1 (DSM 21510) and *D. kuznetsovii* (DSM 6115). *T*. sp. strain X514 (ATCC BAA‐938) was obtained from the American Type Culture Collection (Manassas, VA, USA). *G. stearothermophilus* NUB3621 (BGSC* 9A5) was obtained from the Bacillus Genetic Stock Center (Columbus, OH, USA). The *C. thermocellum* AdhE gene was PCR‐amplified from *C. bescii* heterologous expression strain JWCB032 (Chung *et al*., 2014).

### Plasmid construction and DNA manipulation

A linear transformation construct was prepared for deletion of the *P. furiosus* AOR gene (PF0346). Flanking regions of ~0.5 kb upstream and downstream of *aor* were PCR‐amplified from *P. furiosus* genomic DNA and combined by splice‐overlap extension PCR (SOE‐PCR, (Horton *et al*., 1989)) with a PCR‐amplified pop‐out P_*gdh*_ *pyrF* marker cassette (Keller *et al*., 2013) positioned between the flanking regions.

A vector was constructed for the insertion of heterologous expression cassettes at a location identified here as ‘Genome Region 4’, between convergent genes PF0738.1n and PF0739. The insertion site for Genome Region 4 is between nucleotides 734567–734568 in the *P. furiosus* DSM 3638 genome (AE009950). This position has little to no transcriptional activity as determined from analysis of tiling array data (Yoon *et al*., 2011). SOE‐PCR was used to combine ~0.5 kb flanking regions to Genome Region 4 with the intervening pop‐out marker cassette. This fragment was cloned into pJHW006 (Lipscomb *et al*., 2011) using NheI and NdeI restriction sites to make plasmid pGL008. AdhE gene sequences of *G. thermoglucosidasius*,* G. stearothermophilus*,* T. celere*,* A. flavithermus*,* D. kuznetsovii and C. thermocellum* were PCR‐amplified and combined with the *slp* promoter (P_*slp*_) (Keller *et al*., 2013) and the pGL008 plasmid backbone via Gibson Assembly (Gibson *et al*., 2009; Merryman and Gibson, 2012), (NEB) to make plasmids pGL085, pGL086, pGL087, pGL088, pGL089 and pGL107 respectively. These plasmid constructs were sequence‐verified. The Te*adhE*,* TxadhE* and Tx*adhA* genes were PCR‐amplified and combined with flanking regions and the pop‐out marker cassette via SOE‐PCR, and the PCR constructs were used for transformation of *P. furiosus*. PCR constructs for construction of the mutant AdhE strains were amplified from genomic DNA of Tx‐AdhE and Te‐AdhE strains, each in two segments, using primers to introduce the desired mutation in the AdhE gene (G532D for Te*adhE* and G557D for Tx*adhE*). The two segments for each were joined by SOE‐PCR with the overlap region containing the introduced mutation.

### P. furiosus strain construction

The Δ*aor* PCR construct was used to transform *P. furiosus* COM1 (Δ*pyrF*) as previously described (Lipscomb *et al*., 2011). Transformant colonies were screened by PCR using primers amplifying from outside the flanking regions used for homologous recombination. The strain was colony purified on solid defined medium lacking uracil (Lipscomb *et al*., 2011), the *aor* deletion was sequence‐verified, and the strain was designated MW616. To construct a markerless Δ*aor* genetic acceptor strain, loss of the pop‐out marker cassette was selected for on solid defined medium containing 2.5 mM 5‐FOA and 20 μM uracil as previously described (Lipscomb *et al*., 2011; Keller *et al*., 2013). Isolates were screened by PCR to verify marker loss and were further purified on solid defined medium with 20 μM uracil. A markerless Δ*aor* strain was designated MW252. MW252 was transformed as previously described (Lipscomb *et al*., 2011) with linearized plasmids pGL085, pGL086, pGL087, pGL088, pGL089 and pGL107 as well as PCR constructs containing P_*slp*_TeAdhE, P_*slp*_TxAdhE, P_*slp*_TeAdhEA, P_*slp*_Te*adhE*(G532D) and P_*slp*_Tx*adhE*(G557D). Transformants were screened by PCR with primers outside the homologous flanking regions to Genome Region 4, and strains were colony purified on solid defined medium lacking uracil. For strains that were transformed with PCR constructs, the inserted region was sequence‐verified. Final strains were designated as indicated in Table 2.

### Growth of archaeal and bacterial strains

*Pyrococcus furiosus* strains were cultured anaerobically in a sea water‐based medium as previously described (Keller *et al*., 2015) containing 5 g l^−1^ maltose, 5 g l^−1^ yeast extract and 0.5 μg l^−1^ riboflavin (referred to as YM5 medium). Cultures were grown at 95°C until they reached 1 × 10^8^ cells ml^−1^ at which point the temperature was decreased to 65°C. For metabolite analysis, 50 ml cultures were grown in 150 ml serum bottles. Samples were taken at the time of the temperature switch and 24, 56 and 90 h after the switch. For enzymatic analysis, 250 ml cultures were grown in 1 l anaerobic bottles and the cultures were harvested after 40 h by centrifugation at 6000 × g for 10 min. For the bacterial wild‐type strains of *T*. sp. X514 and *T. ethanolicus*, 250 ml cultures in 1 l anaerobic bottles were grown in *C. bescii* media (Yang *et al*., 2010) with the addition of 5 g l^−1^ xylose. Cultures were incubated at 65°C for 16–24 h to 1 × 10^8^ cells ml^−1^ before harvesting by centrifugation at 6000 × g for 10 min. *C. thermocellum* cell pellets were obtained from David Mulder and Paul King at the National Renewable Energy Laboratory (NREL).

### In vivo ethanol and acetate analysis

Strains were grown in triplicate as described above for *in vivo* ethanol quantification. A deep‐well 96‐well plate was used to collect the 1 ml samples. These plates were centrifuged at 4000 × g to pellet the cells. A multichannel pipette was used to transfer 200 μl of supernatant to vial inserts containing 5 μl of 88% formic acid. The inserts were assembled into vials and capped. The samples were then analysed via GC‐FID with an identical method as previously described (Keller *et al*., 2015). Maltose consumption was estimated from quantified amounts of ethanol and acetate, with the assumption that these are the major carbon end‐products after glycolysis.

### Cell extract preparation

Cell extracts of *P. furiosus* strains and bacterial wild‐type strains were prepared under strict anaerobic conditions in an anaerobic chamber (Coy Laboratories). Cell pellets were suspended in 0.5–1.25 ml 100 mM MOPS, pH 7.0, containing 5 μM FeCl_2_ (Zheng *et al*., 2015). Lysozyme was added to the suspended bacterial cells, and these were incubated at 25°C for 20 min. The cell lysate was then centrifuged at 10 000 × g for 10 min, and the supernatant was transferred to a vial which was sealed and stored at 4°C. The protein concentration of the extracts was measured using Bradford reagent (Bio‐Rad, Hercules, California, U.S.A.).

### In vitro analysis of ALDH, ADH and AOR activities

All reactions were carried out in sealed anaerobic cuvettes using 25 mM MOPS, pH 7.0 containing cell extract at a final concentration of 0.2 mg ml^−1^. ADH and ALDH activities were measured as 65°C using NADPH or NADH at a concentration of 0.2 mM. After the addition of either 0.5 mM acetyl‐CoA for ALDH or 10 mM acetaldehyde for ADH, the rate of NAD(P)H oxidation was measured at 340 nm and calculated using a molar absorptivity of 6220 M^−1^ cm^−1^. Native *P. furiosus* ADH activity was measured at 80°C in 100 mM EPPS, pH 8.0, using 0.5 mM NADP and 50 mM ethanol. AOR activity was also measured at 80°C in 100 mM EPPS, pH 8.0, using the redox dye benzyl viologen (BV, 1 mM) as the electron acceptor. The AOR reaction was initiated by the addition of 10 mM acetaldehyde, and the rate of BV reduction was measured at 600 nm and calculated using a molar absorptivity of 10 000 M^−1^ cm^−1^.

## Conflict of interest

None declared.

## Supporting information


**Table S1.** Amino acid sequence identity (%) among the eight AdhE proteins.^a^
**Fig. S1.** Deletion of *aor* does not affect growth of *P. furiosus* and abolishes 80% of aldehyde oxidoreductase activity.
**Fig. S2.** Nucleotide specificity mutation does not improve ethanol yield.
