# Supplementary material for: Ethanol production by the hyperthermophilic archaeon Pyrococcus furiosus by expression of bacterial bifunctional alcohol dehydrogenases
Source: Microb Biotechnol. 2017 Feb 14;10(6):1535–45. doi: 10.1111/1751-7915.12486 (PMC5658578; doi:10.1111/1751-7915.12486)
Supplement: Supplementary file 1 — Table S1. Amino acid sequence identity (%) among the eight AdhE proteins.a Fig. S1. Deletion of aor does not affect growth of P. furiosus and abolishes 80% of aldehyde oxidoreductase activity. Fig. S2. Nucleotide specificity mutation does not improve ethanol yield. [file MBT2-10-1535-s001.pdf]

Microbial Biotechnology

(MICROBIO-2016-167-RA)

Supporting Information

Ethanol Production by the Hyperthermophilic Archaeon *Pyrococcus furiosus* by Expression of Bacterial Bifunctional Alcohol Dehydrogenases

Matthew W. Keller[1†], Gina L. Lipscomb[1†], Diep M. Nguyen[1], Alexander T. Crowley[1], Gerrit J. Schut[1], Israel Scott[1], Robert M. Kelly[2] and Michael W. W. Adams[1*]

[1]Department of Biochemistry & Molecular Biology, University of Georgia,

Athens, GA 30602

[2]Department of Chemical and Biomolecular Engineering,

North Carolina State University, Raleigh, NC 27695

*Address correspondence to adamsm@uga.edu

[†]M.W.K. and G.L.L. contributed equally to this work.

Table S1. Amino acid sequence identity (%) among the eight AdhE proteins [a]

	Te	Tx	Gs	Gt	Af	Ct	Tc	Dk
**Te**	100	97	67	68	68	52	54	65
**Tx**	97	100	68	68	68	52	54	65
**Gs**	67	68	100	97	88	51	53	63
**Gt**	64	68	97	100	89	51	54	63
**Af**	68	68	88	89	100	51	56	64
**Ct**	52	52	51	51	51	100	71	47
**Tc**	54	54	53	54	56	71	100	49
**Dk**	65	65	63	63	64	47	49	100

[a] Organism abbreviations are as follows: Te, *Thermoanaerobacter ethanolicus* JW200; Tx, *Thermoanaerobacter* sp. X514; Gs, *Geobacillus stearothermophilus* NUB3621; Gt, *Geobacillus thermoglucosidasius* NBRC 107763 Af, *Anoxybacillus flavithermus* WK1; Ct, *Clostridium thermocellum*; Tc, *Thermobrachium celere* DSM 8682; Dk, *Desulfotomaculum kuznetsovii* DSM 6115.

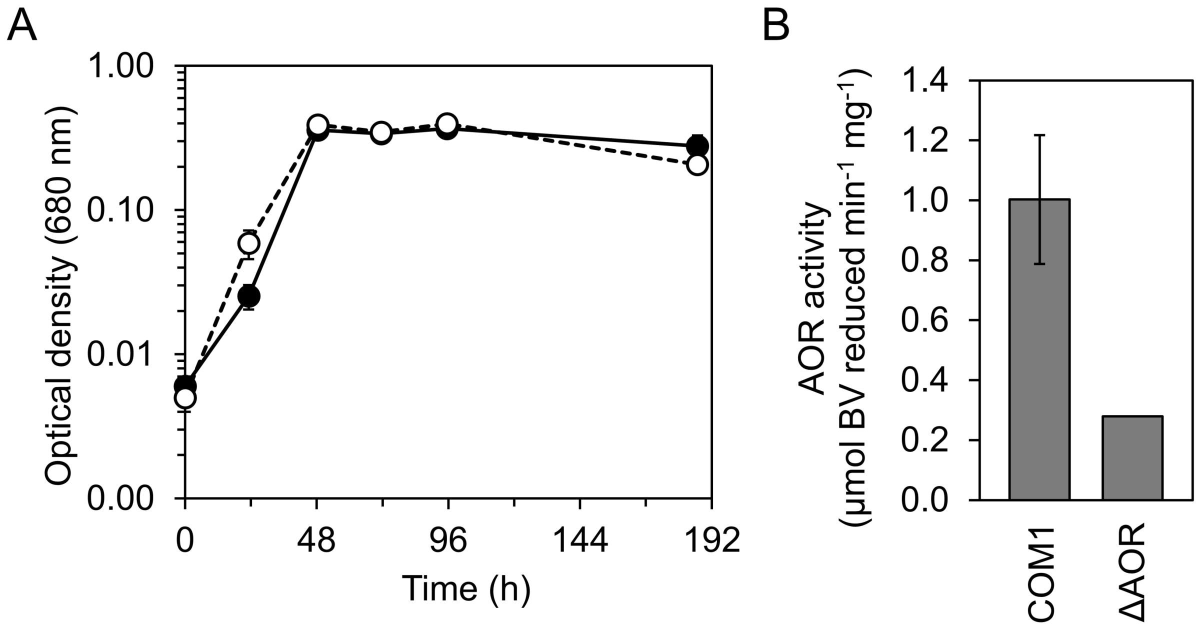

Figure S1. Deletion of *aor* does not affect growth of *P. furiosus* and abolishes 80% of aldehyde oxidoreductase activity. **A.** MW004 (Δ*pyrF*::*pyrF*, black line with closed circles) and MW616 (Δ*pyrF* Δ*aor*::P_{gdh}*pyrF*, dashed line with open circles) strains were grown at 72°C for 72 h and then at 65°C up to 8 d in YM medium containing a total of 2 g L^{-1} yeast extract. Error bars represent S.D., *n* = 3. **B**. Aldehyde oxidoreductase (AOR) activity in cell extracts of COM1 (Δ*pyrF*) and ΔAOR(Δ*pyrF* Δ*aor*).

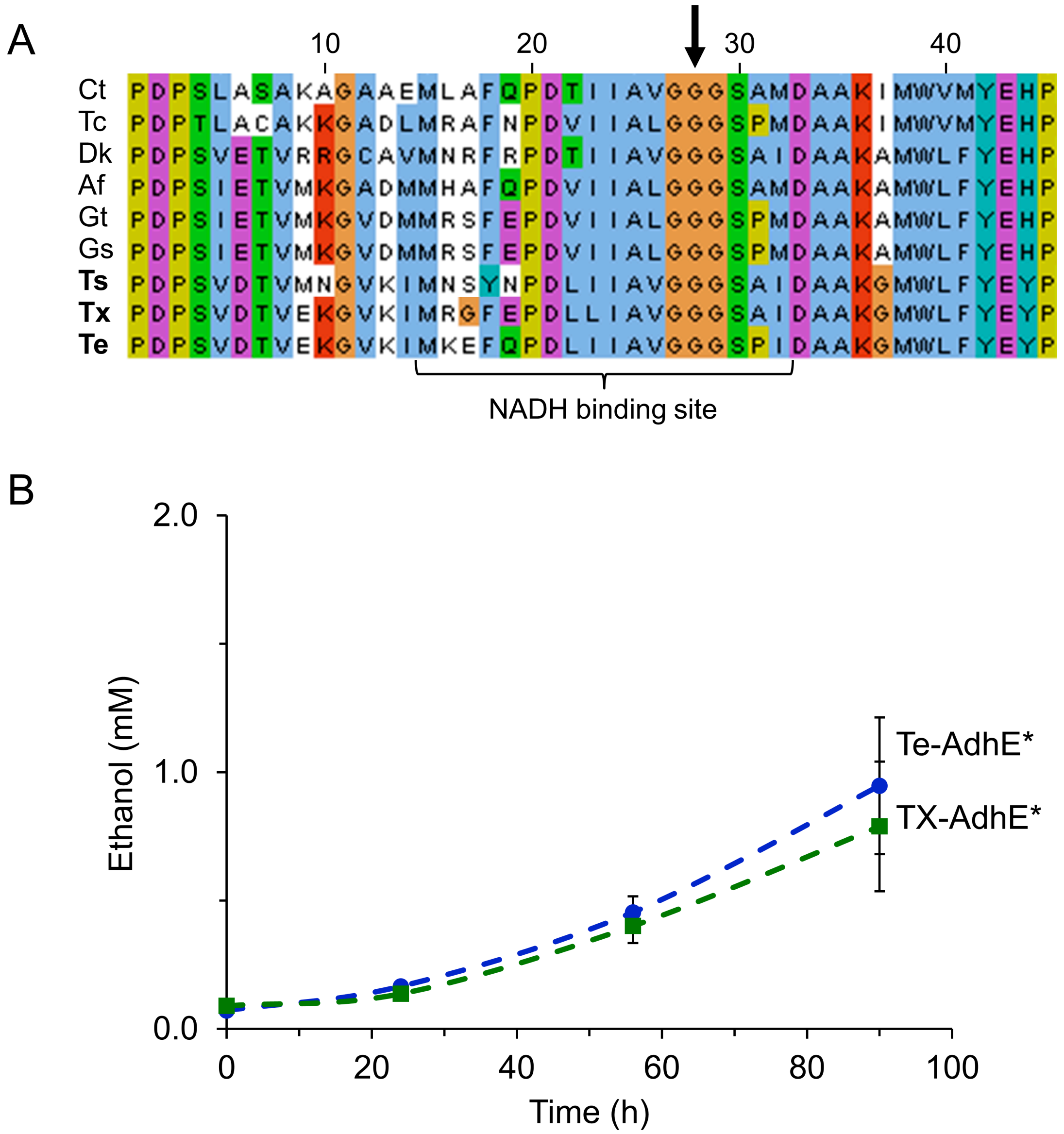

Figure S2. Nucleotide specificity mutation does not improve ethanol yield. **A.** Multiple alignment of ADH domain NADH binding site of selected AdhEs with *T. saccharolyticum* AdhE. Alignment performed using Clustal Omega (Sievers, et al., 2011) and visualized with Jalview (Clamp, et al., 2004). Location of NADH binding site is indicated with a bracket and the position of the G→D mutation (G544D in *T. saccharolyticum*) is indicated with an arrow. Organism abbreviations are as follows: Ct, *Clostridium thermocellum*; Tc, *Thermobrachium celere* DSM 8682; Dk, *Desulfotomaculum kuznetsovii* DSM 6115; Af, *Anoxybacillus flavithermus* WK1; Gt, *Geobacillus thermoglucosidasius* NBRC 107763; Gs, *Geobacillus stearothermophilus* NUB3621; Ts, *T. saccharolyticum*; Tx, *Thermoanaerobacter* sp. X514; Te, *Thermoanaerobacter ethanolicus* JW200. **B.** Ethanol production over time for Te-AdhE and Tx-AdhE, grown in closed serum bottles with 50 mL YM5 medium to mid-log phase at 95°C and then incubated at 65°C for 90 h. Error bars represent S.D., *n* = 3.

References

1 Sievers, F., Wilm, A., Dineen, D., Gibson, T.J., Karplus, K., Li, W., et al. (2011) Fast, scalable generation of high-quality protein multiple sequence alignments using Clustal Omega, *Molecular Systems Biology* **7**: 539-539.

2 Clamp, M., Cuff, J., Searle, S.M., and Barton, G.J. (2004) The jalview java alignment editor, *Bioinformatics* **20**: 426-427.
